# Phenolic Compounds in Extra Virgin Olive Oil Stimulate Human Osteoblastic Cell Proliferation

**DOI:** 10.1371/journal.pone.0150045

**Published:** 2016-03-01

**Authors:** Olga García-Martínez, Elvira De Luna-Bertos, Javier Ramos-Torrecillas, Concepción Ruiz, Egle Milia, María Luisa Lorenzo, Brigida Jimenez, Araceli Sánchez-Ortiz, Ana Rivas

**Affiliations:** 1 Faculty of Health Sciences, University of Granada, Avda de la Ilustración s/n, 18071, Granada, Spain; 2 Faculty of Medicine and Surgery, University of Sassari, Piazza Universitá 21, 07100, Sassari, Italy; 3 Department of Nutrition and Food Science, Faculty of Pharmacy, University of Granada, Campus de Cartuja s/n, 18071, Granada, Spain; 4 Agricultural Research Training Centre, Ministry of Agriculture and Fisheries, Ctra. Cabra-Doña Mencía, Km. 2.5, 14940, Cabra, Córdoba, Spain; 5 Agricultural Research Training Centre, Ministry of Agriculture and Fisheries, Ctra. Bailen-Motril, 23620, Km. 18,5, Mengibar, Jaén, Spain; University of Palermo, ITALY

## Abstract

In this study, we aimed to clarify the effects of phenolic compounds and extracts from different extra virgin olive oil (EVOO) varieties obtained from fruits of different ripening stages on osteoblast cells (MG-63) proliferation. Cell proliferation was increased by hydroxytyrosol, luteolin, apigenin, *p-*coumaric, caffeic, and ferulic acids by approximately 11–16%, as compared with controls that were treated with one vehicle alone, while (+)-pinoresinol, oleuropein, sinapic, vanillic acid and derivative (vanillin) did not affect cell proliferation. All phenolic extracts stimulated MG-63 cell growth, and they induced higher cell proliferation rates than individual compounds. The most effective EVOO phenolic extracts were those obtained from the Picual variety, as they significantly increased cell proliferation by 18–22%. Conversely, Arbequina phenolic extracts increased cell proliferation by 9–13%. A decline in osteoblast proliferation was observed in oils obtained from olive fruits collected at the end of the harvest period, as their total phenolic content decreases at this late stage. Further research on the signaling pathways of olive oil phenolic compounds involved in the processes and their metabolism should be carried out to develop new interventions and adjuvant therapies using EVOO for bone health (i.e.osteoporosis) in adulthood and the elderly.

## Introduction

Bone health is a major public health issue. Osteoporosis is a disease that affects many millions of people around the world and will take on increasing significance as people live longer and the world´s population continues to increase in number [1]. Although nutrition is only one of the many factors that influence bone mass and fragility fractures, it is of particular importance to bone health because it is modifiable.

In Europe, conspicuous differences are encountered in the severity of osteoporosis, the lowest incidence being reported in the Mediterranean area. Among the environmental factors underlying this difference is the traditional Mediterranean diet, rich in fruit and vegetables consumption, with a high intake of olive oil [2, 3]. The biological properties of the phenolic compounds found in Extra Virgin Olive Oil (EVOO) have been extensively studied [4]. However, despite the myriad of potential health benefits of olive oil phenolic compounds, there are only few data relating their possible preventive effect on osteoporosis [5–9]. It has been described that the consumption of olives, olive oil, and oleuropein and hydroxytyrosol–the main phenolic compounds in leaves and fruit oil respectively–can prevent the loss of bone mass in animal and cell models [7]. Much less is known about the effects of other olive oil phenolic compounds on bone health. Recently, we have demonstrated that the phenolic fractions of Sicilian EVOO induced osteoblast cell growth using the human MG-63 osteosarcoma cell line [9].

Osteoblasts are bone-forming cells derived from undifferentiated pluripotent mesenchymal cells. The formation of bone involves a complex series of events that include osteoprogenitor cell proliferation and differentiation, and eventually result in the formation of a mineralized extracellular matrix. Numerous cytokines, hormones, and growth factors control bone formation by regulating osteoblast cell proliferation and differentiation. It has been demonstrated that phenolic compounds may modulate osteoblast cell functions [10, 11]. However, to our knowledge, there is no information available on the ability of olive oil phenolic compounds or olive oil extracts to regulate cultured osteoblast proliferation.

The most important phenolic compounds that have been identified on EVOO may be divided into different groups such as phenolic acids, phenolic alcohols, secoiridoids, lignans, and flavones [12].The phenolic content of EVOO is influenced by several factors: the harvest date–during which the qualitative and quantitative composition of the phenolic substances undergoes sharp fluctuations-[13], industrial processing techniques, and storage and preservation methods [14]. Therefore, the aims of this study were, firstly, to investigate the effects of EVOO phenolic compounds on osteoblast cell growth, using the human MG-63 osteosarcoma cell line. Secondly, we investigated the influence of olive variety and ripening degree on the osteoblastic cell proliferation effect of EVOO phenolic extracts. To such purpose, we sampled four monovarietal EVOO obtained from fruits at three different ripening stages.

## Material and Methods

### Chemicals

Standards of apigenin, luteolin, hydroxytyrosol, tyrosol, vanillin, caffeic, *p*-coumaric, ferulic, sinapic and vanilic acids were purchased from Sigma-Aldrich (St. Louis, MO); (+)-pinoresinol was from Arbo-Nova (Turku, Finland), and oleuropein was acquired from Extrasynthese (Genay, France). Stock solutions of phenolic compounds were prepared in methanol and stored at -20°C. All the solvents used were of analytical or HPLC grade (Sigma-Aldrich). Water was of Milli-Q quality (Millipore Corp, Bedford, MA, USA).

### Olives

Olive fruits were sampled from olive trees of the cultivars Picual, Arbequina, Picudo and Hojiblanca grown in the experimental farm at the Agricultural Research Training Centre in Cabra (Cordoba, Southern Spain). Ten adult 27 year-old olive trees of the varieties Picual and Hojiblanca and 16 year-old olive trees of the Arbequina and Picudo varieties, spaced 12x12 m^2^, were identified and carefully marked. Olive samples were hand-picked, at the beginning, middle, and end of the harvest from different olive varieties. The olive ripening index was determined according to the method proposed by the International Olive Oil Council (IOOC), based on olive skin and pulp colour [15]. Only healthy fruits without any kind of infection or physical damage were processed.

### Oil Samples

Extra virgin olive oil (EVOO) samples were obtained using an Abencor analyzer (Abengoa S.A., Sevilla, Spain); this system reproduces the industrial process at laboratory scale and consists of three basic elements: hammer mill, thermobeater, and pulp centrifuge [16]. The EVOO obtained was decanted and stored in amber glass bottles at 4°C in darkness without headspace until analysis.

### Sample Preparation

To isolate the phenolic fraction of olive oils, we used the method proposed by the IOOC [2, 17, 18]. Briefly, the analytical methodology combines olive oil extraction with methanol/water (80/20), ultrasonic bath for 15 min at ambient temperature and centrifuge at 5000 rev/min for 25 min. After that, an aliquot of the supernatant phase is filtered through a 5 ml plastic syringe using a Millex®-HV PVDF 0.45 μm filter (Millipore Corp, Billerica, MA, USA). Extractions were replicated three times, and phenolic extracts were stored at -20°C until analysis.

### Total Phenol Measurements

Phenolic extracts were subsequently quantified by HPLC using an UV-Vis detector following IOOC’s methodology [17]. The content of total phenols is expressed in mg/kg of tyrosol. HPLC analyses were performed with a Varian ProStar (Walnut Creek, CA, USA), equipped with a binary pump delivery system, and a Varian 230 UV-Vis detector. A Waters Spherisorb® 5 mm ODS2 column, 5 μm, 4.6 cm x 200 mm (Marlborough, MA, USA) was used.

### UPLC-TOF- MS Analyses of Individual Phenolic Compounds

We used ultra performance liquid chromatography coupled to time-of-flight mass spectrometry (UPLC-TOF-MS) analysis to detect and measure individual phenolic compounds in prepared phenolic extracts. The UPLC system consisted of an AcQuity TM UPLC equipped with a binary pump system (Waters, Milford, MA, USA) using an AcQuity UPLC TM BEH C18 column (1.7 μm, 100 mm x 2.1 mm i.d.) from Waters. The UPLC-TOF-MS methodology was describes elsewhere [18]. The identification of phenolic compounds was carried out by comparing both, retention times and MS spectral data from olive oil samples and standards.

### Cell Culture

The human osteosarcoma cell line MG-63 was purchased from American Type Cultures Collection (ATCC, Manassas, VA) and maintained in Dulbecco’s Modified Eagle Medium (DMEM; Invitrogen Gibco Cell Culture Products, Carlsbad, CA) with 100 IU/ml penicillin (Lab Roger SA, Barcelona, Spain), 50 μg/ml gentamicin (Braum Medical SA, Jaen, Spain), 2.5 μg/ml amphotericin B (Sigma, St Louis, MO, USA), 1% glutamine (Sigma, St Louis, MO, USA), 2% HEPES (Sigma, St Louis, MO, USA), and supplemented with 10% fetal bovine serum (FBS) (Gibco, Paisley, UK). Cultures were kept at 37°C in a humidified atmosphere of 95% air and 5% CO2. Cells were detached from the culture flask with a solution of 0.05% Trypsin (Sigma, St Louis, MO, USA) and 0.02% ethylenediaminetetraacetic acid (EDTA) (Sigma, St Louis, MO, USA) and then washed and suspended in complete culture medium with 10% FBS. Prior to the beginning of each experiment, all cells were grown in estrogen-free media (DMEM without red phenol) for at least 24 hours.

### Cell Proliferation Assay

The cell proliferation methodology was described elsewhere [19, 20]. Briefly, proliferation was determined with an MTT method (Sigma-Aldrich Chemie). This is a colorimetric method that measures the chemical reduction of MTT (3-(4,5-Dimethylthiazol-2-yl)-2,5-diphenyltetrazolium bromide) into formazan, which is directly proportional to the number of viable cells in the tested culture. Osteoblasts were seed at 1 x 10^4^ cells/ml per well into a 96-well plate (Falcom, Becton Dickinson Labware, New Jersey) in estrogen-free culture medium without FBS and cultured at 37°C in a humidified atmosphere of 95% air and 5% CO2 for 24 hours. Then, the media was replaced with DMEM containing different concentrations of treatment compounds: phenolic compounds at 10^−5^, 10^−6^, 10^−7^, 10^−8^ and 10^−9^ M and prepared phenolic extracts dissolved in fresh culture medium at concentrations of 0.001, 0.0001 and 0.00001%. All experiments included cells incubated under the same conditions without treatment compounds and incubated cells with 0.001% of methanol as an internal control. Three separate experiments were performed for each treatment and at least every experiment was performed in triplicate. On completion of treatment, the media were replaced with DMEM, without phenol-red, containing 0.5 mg/ml MTT (Sigma, St Louis, MO, USA) and incubated for 4 hours. Cellular reduction of the tetrazolium ring of MTT resulted in the formation of a dark-purple water-insoluble deposit, formazan crystals. After incubation, the media was aspired and DMSO was added to dissolve formazan crystals. Absorbance was measured at 570 nm with a spectrophotometer (Sunrise TM, Tecan, Männedorf, Switzerland). Cell proliferation percentages (% > control) were calculated as related to cell cultures treated with methanol alone (controls).

### Statistical analysis

Statistical analysis was performed using SPSS (v. 15.0, Statistical Package for the Social Sciences, Chicago, IL). Numerical data on cell proliferation are expressed as the mean of three independent experiments; statistically significant differences were analyzed by Student´s t-test. Significance of differences at a 5% level among phenolic compounds means was determined by one-way ANOVA, using Tukey´s test. The results for phenolic compounds are the average of at least three repetitions. Principal components analysis (PCA) was applied to data. PCAs were carried out for all raw data processed with a cross-validation method. Data were auto-scaled before analysis.

## Results and Discussion

### Effect of olive oil phenolic compounds on MG-63 osteoblast cells proliferation

Nutritional and pharmacological factors are needed to prevent bone loss with aging. In this study, we aimed to clarify the potential effects of olive oil phenolic compounds on osteoblast proliferation. MG-63 osteoblast cells were cultured in the presence of phenolic-compound methanolic dilutions in fresh culture medium. The MG63 human osteosarcoma line was selected as osteoblast model in this study because it is the most widely used cell line in studies on the effects of drug or other treatments on the osteoblast [21, 22], although the limitations of studying a tumor line should be borne in mind, as the differentiation pattern of MG63 may differ from that of real osteoblasts. Twelve olive oil phenolic compounds were evaluated. The highest solvent concentration in culture media (0.001%, v/v methanol) had no significant effects on cell proliferation. Treatments with phenolic compounds (10^−4^ to 10^-3^M) decreased dramatically cell proliferation (data not shown), suggesting that higher concentrations of these compounds were cytotoxic in MG-63 cells.

There were remarkable differences in the ability of individual phenolic compounds to stimulate osteoblast proliferation (Table 1). Thus, concentrations as high as 10^−6^ M were needed to significantly increase cell proliferation when MG-63 cells were cultured in the presence of hydroxytyrosol. Exposure of osteoblast cells to hydroxytyrosol increased the number of cells by approximately 11% in 24 h, as compared with control cultures that were treated with one vehicle alone. Conversely, concentrations lower than 10^-6^M did not have any significant effect on cells. The underlying mechanism of the effect of hydroxytyrosol on MG-63 cells needs to be elucidated. However, despite of these results, we can not affirm that the polyphenols in general are mitogenic for osteoblasts. Moreover, other *in vitro* studies have shown that hydroxytyrosol stimulates osteoblastic cell activity. These studies have used another cell line (MC3T3 -E1 cells) and obtained similar results to ours with the MG63 cell line [5].

**Table 1:** Proliferation of MG-63 cells with treatment of olive oil phenolic compounds.

Treatment	Concentration (Molar)	Proliferation^a^	t	p
Control		100		
Hidroxytyrosol	10^−5^	107.34 ± 5.02	-2.529	0.065
	10^−6^	111.42 ± 7.31	-2.477	0.027
	10^−7^	101.47 ±13.76	-0.238	0.815
	10^−8^	100.96 ± 8.31	-0.456	0.651
Tyrosol	10^−5^	95.25 ± 17.84	0.461	0.669
	10^−6^	96.10 ± 15.50	0.583	0.569
	10^−7^	93.02 ± 17.43	0.962	0.352
	10^−8^	91.89 ± 11.38	1.462	0.166
Caffeic acid	10^−5^	115.9 ± 3.50	-7.85	0.001
	10^−6^	116.31 ± 9.61	-2.469	0.023
	10^−7^	115.31 ± 8.78	-0.100	0.022
	10^−8^	111.58 ± 6.20	-0.906	0.050
Vanillic acid	10^−5^	98.2 ± 14.70	0.212	0.852
	10^−6^	97.15 ± 11.96	0.465	0.649
	10^−7^	96.83 ± 6.37	0.640	0.533
	10^−8^	97.89 ± 13.37	0.326	0.749
Vanillin	10^−5^	103.36 ± 11.05	-0.528	0.626
	10^−6^	103.50 ± 8.54	-0.681	0.507
	10^−7^	94.77 ± 10.55	0.979	0.344
	10^−8^	97.49 ± 8.62	0.512	0.616
p-coumaric acid	10^−5^	112.30 ± 5.05	-4.218	0.013
	10^−6^	112.08 ± 9.18	-1.802	0.050
	10^−7^	113.28 ± 7.32	-1.821	0.049
	10^−8^	111.47 ± 6.03	-0.193	0.048
Ferulic acid	10^−5^	115.10 ± 2.90	-4.019	0.001
	10^−6^	115.69 ± 5.05	-3.363	0.002
	10^−7^	113.74 ± 4.61	-2.958	0.006
	10^−8^	112.13 ± 4.27	-2.617	0.014
Sinapic acid	10^−5^	94.57 ± 11.02	0.853	0.442
	10^−6^	94.61 ± 10.63	1.073	0.292
	10^−7^	92.90 ± 14.84	1.310	0.200
	10^−8^	103.12 ± 9.36	-0.626	0.529
Oleuropein	10^−5^	100.06 ± 17.46	-0.007	0.995
	10^−6^	99.99 ± 12.25	0.001	0.999
	10^−7^	91.64 ± 13.73	1.319	0.210
	10^−8^	106.51 ± 11.07	-1.192	0.253
Luteolin	10^−5^	113.99 ± 3.02	-8.024	0.001
	10^−6^	114.29 ± 3.07	-3.107	0.004
	10^−7^	110.78 ± 2.83	-2.348	0.026
	10^−8^	115.80 ± 5.73	-3.366	0.002
(+)-pinoresinol	10^−5^	106.02 ± 5.01	-2.081	0.106
	10^−6^	106.65 ± 7.24	-1.956	0.060
	10^−7^	95.04 ± 8.83	0.790	0.436
	10^−8^	97.23 ± 6.52	0.782	0.452
Apigenin	10^−5^	110.43 ± 1.56	-1.56	0.001
	10^−6^	111.01 ± 9.87	-0.260	0.020
	10^−7^	110.11 ± 6.40	-2.556	0.016
	10^−8^	109.83 ± 5.16	-2.625	0.014

a. Values are mean ± SD (% control) of three separate experiments performed, and at least every experiment was performed in triplicate. Statistically significant differences were analyzed by Student´s t-test. Statistical significance was recognized at p<0.05.

Among olive oil polyphenols, hydroxytyrosol has the strongest antioxidant effects [4]. Oxidative stress resulting in increased levels of intracellular reactive oxygen species has been reported to suppress bone metabolism. It has been recently demonstrated that hydroxytyrosol effectively decreases H_2_O_2_ levels in MC3T3-E1 osteoblastic cells [5]. In addition, H_2_O_2_ has been reported to suppress differentiation markers–such as alkaline phosphatase activity–, type I collagen gene expression, and osteoblastic cell mineralization [23].

Treatment of osteoblast cells with olive oil phenolic acids increased the number of cells approximately by 12–16% as compared with controls. The investigated phenolic acids affect MG-63 cells differently. Cell proliferation was increased by *p-*coumaric, caffeic, and ferulic acids by approximately 12–16%, as compared with controls in the range of 10^−5^ to 10^−9^ M, while sinapic, vanillic acid and derivative (vanillin) did not affect cell proliferation (Table 1). Proliferation increase was not dose-dependent, probably due to plateau reached at the lowest concentration. Researchers have shown the phenolic compounds in different vegetable species can modulate the functions of osteoblastic cells, including their proliferative capacity and maturation, by increasing alkaline phosphatase activity and the deposit of calcium ions in the extracellular matrix [5, 10]. The mechanisms proposed to underlie these changes in osteoblastic activity include the modification of osteoblast function by certain phenolic compounds via the modulation of different transcription factors, e.g., Cbfa1/Runx2, and bone morphogenetic proteins, e.g., osterix and osteocalcin; these are all essential molecules to induce osteoblast differentiation, which in turn may activate genes involved in the bone remodelling process [10, 24, 25]

The effect of phenolic acids on MG-63 proliferation could be added to the list of positive effects of these acids such us their anti-inflamatory, antioxidant, antimutagenic, anticarcinogenic, and body weight activities [26]. There is also growing evidence both *in vitro* and *in vivo* studies that natural phenolic acids may favorably affect the skeletal system. This effect has been attributed to the inhibition of bone resorption and stimulation of bone formation [27, 28]. In addition, Chen et al. [29] showed that phenolic acids derived from the breakdown of blueberries polyphenols appear in the serum following consumption and stimulate osteoblast differentiation through Wnt signaling, indicating their potential in the prevention of bone loss.

A possible mechanism of phenolic acids on osteoblast to be considered is their binding to estrogen receptors, which are found in osteoblastic cells [30]. The presence of 17 β-estradiol has been reported to induce a significant increase in osteoblastic cell proliferation, DNA and protein content, and alkaline phosphatase activity [31]. There are sparse experimental data that phenolic acids may have some *in vitro* estrogenic activity. Ferulic acid estrogen receptor-dependently stimulated proliferation of human breast cancer cells in a concentration and estrogen receptor-dependent manner [32]. On the other hand, caffeic acid has been reported to increase the proliferation of MCF7 cells [33]. In addition, there are also some data available on the *in vivo* estrogenic effects of phenolic acids. In ovariectomized rats, ferulic acid, caffeic acid and, to a lesser extent, *p*-coumaric acid increased serum estradiol levels [34].

The effect of oleuropein–a polyphenol belonging to the secoiridoid class found in olives and their derivatives–on human osteosarcoma MG-63 proliferation was also investigated. Oleuropein did not induce MG-63 osteosarcoma cell proliferation. To our knowledge, there are no data available on the effect of oleuropein on human osteoblasts. However, it has been described that the consumption of oleuropein can prevent the loss of bone mass in a rat model of bone mass loss associating ovariectomy and acute inflammation [35].It is interesting to note that this compound was not able to affect bone mineral density in ovariectomised rats when inflammation was not induced [7, 36], which suggests that oleuropein may exert its bone-sparing effect by modulating inflammation rather than acting directly on bone metabolism [37].

Flavonoids and Lignans–groups of phenolic compounds abundant in plants–are known to have many beneficial biological effects including anti-inflammatory, antioxidant and estrogenic activity. In this sense, (+)-pinoresinol–a major component in the lignan phenolic fraction of olive oil–did not have any effect on cell proliferation. On the contrary, in the rat osteosarcoma cell line UMR106, pinoresinol shows stimulating effects both on UMR 106 cell proliferation and alkaline phosphatase activity [38]. Luteolin and apigenin–which are olive oil flavonoids–increased cell proliferation by 11–15% in all tested concentrations. It has been shown that luteolin inhibited the bone resorptive activity of differentiated osteoclasts [39]; additionally, luteolin has an anabolic effect, as it increases collagen synthesis, alkaline phosphatase (ALP) activity, and osteocalcin secretion, and it inhibits 3-morpholinosydnonimine-stimulated production of proinflammatory mediators in osteoblastic MC3T3-E1 cells *in vitro* [40]. Also, oral administration of luteolin (5 and 20 mg/kg per day) to ovariectomised mice caused significant increase in bone mineral density and bone mineral content of trabecular and cortical bones in the femur [39]. It should be pointed out that the mechanism of action of flavonoids on bone has been partly attributed to estrogen action [41].

### Identification and quantification of phenolic compounds in EVOO

We identified and quantified the phenolic compounds in phenolic extracts obtained from EVOOs Picual, Arbequina, Hojiblanca and Picudo used in MG-63 cell proliferation assays. Mass spectral data and retention time are reported for all identified compounds (Table 2). Table 3 shows the mean values for the total and individual phenolic compounds detected in oils of the varieties Picual, Hojiblanca, Arbequina, and Picudo at three different ripening stages. In all varieties, total phenol content decreased during ripening, in accord with the results obtained by other authors in different olive oil varieties [42]. The main phenolic compounds found in the EVOOs under study were tyrosol and hydroxytirosol. The highest amounts of both substances were found at the earliest harvest sample. Our results are in agreement with those of Martinez-Nieto et al. [13], which reported that tyrosol and hidroxytirosol concentrations decreased with increasing olive ripeness in Picual and Arbequina varieties. Caffeic acid was not detected in oils of the Picual, Arbequina and Picudo varieties; *p*-coumaric acid was not detected in Arbequina oils. The phenolic compounds vanillic acid and vanillin were common to all four varieties; only Hojiblanca oils obtained from olives collected at the end of the harvest did not contain vanillic acid.

**Table 2:** Mass spectral data of detected compounds using UPLC-TOF-MS.

Compounds	Molecular formula	Retention Time(min)	M(-)
Hidroxytyrosol	C_8_H_10_O_3_	2.74	153.0552
Tyrosol	C_8_H_10_O_2_	3.49	137.0603
Caffeic acid	C_9_H_8_O_4_	3.90	179.0344
Vanillic acid	C_8_H_8_O_4_	3.93	167.0344
Vanillin	C_8_H_8_O_3_	4.36	151.0395
p-coumaric acid	C_9_H_8_O_3_	4.69	163.0395
Ferulic acid	C_10_H_10_O_4_	5.04	193.0501
Sinapic acid	C_11_H_12_O_5_	5.04	223.0606
Oleuropein	C_25_H_32_O_13_	6.18	539.1765
Luteolin	C_15_H_10_O_6_	7.20	285.0399
(+)-pinoresinol	C_20_H_22_O_6_	7.25	357.1348
Apigenin	C_15_H_10_O_5_	7.30	269.0450

Accurate Mass (M).

**Table 3:** Content of olive oil phenolic compounds (mg/Kg) with regards to olive cultivar and fruit ripening stage.

**COMPOUNDS**	**Picual**	**Picual**	**Picual**	**Arbequina**	**Arbequina**	**Arbequina**
	RI = 0.37	RI = 2.75	RI = 4.67	RI = 0.48	RI = 2.92	RI = 3.87
Hidroxytyrosol	7.50^a^	5.24^b^	4.14^c^	3.03^a^	2.04^b^	1.91^b^
Tyrosol	6.75^a^	5.81^b^	4.27^c^	4.15^a^	2.96^b^	2.46^b^
Caffeic acid	nd	nd	nd	nd	nd	nd
Vanillic acid	0.64^b^	0.80^a,b^	1.09^a^	0.51^b^	0.72^a,b^	1.05^a^
Vanillin	0.15^b^	0.25^b^	0.70^a^	0.40^a^	0.26^a,b^	0.12^b^
p-coumaric acid	0.52^a^	0.54^a^	nd	nd	nd	nd
Ferulic acid	0.03^a^	0.02^a^	0.02^a^	0.24^a^	0.18^b^	0.16^b^
Sinapic acid	nd	nd	nd	nd	nd	nd
Oleuropein	0.01^a^	0.01^a^	nd	0.03	nd	nd
Luteolin	1.66^c^	2.66^b^	2.93^a^	3.26^c^	4.80^b^	6.57^a^
(+)-pinoresinol	1.43^a^	1.22^a,b^	1.02^b^	3.22^a^	2.98^a^	3.03^a^
Apigenin	0.92^a^	0.71^a,b^	0.57^b^	2.12^a^	1.96^a,b^	1.78^b^
Total	648.4^a^	571.3^b^	496.5^c^	320.2^a^	297.4^b^	248.3^c^
**COMPOUNDS**	**Hojiblanca**	**Hojiblanca**	**Hojiblanca**	**Picudo**	**Picudo**	**Picudo**
	RI = 0.51	RI = 1.78	RI = 4.44	RI = 0.88	RI = 1.74	RI = 3.84
Hidroxytyrosol	6.71^a^	5.52^b^	2.51^c^	6.23^a^	5.37^b^	3.09^c^
Tyrosol	6.13^a^	5.77^b^	4.80^c^	5.83^a^	4.85^b^	4.06^b^
Caffeic acid	0.09	nd	nd	nd	nd	nd
Vanillic acid	0.40^b^	0.50^a^	nd	0.57^a^	0.52^a^	0.59^a^
Vanillin	0.64^a^	0.42^b^	0.31^b^	0.22^a^	0.14^b^	0.10^b^
p-coumaric acid	1.03^a^	0.53^b^	nd	0.82^a^	0.50^b^	nd
Ferulic acid	0.09	nd	nd	0.03^a^	0.02^a^	nd
Sinapic acid	nd	nd	nd	nd	nd	nd
Oleuropein	0.02^a^	0.01^a^	0.01^a^	0.02	nd	nd
Luteolin	5.19^b^	5.24^b^	6.21^a^	3.43^c^	5.73^a^	4.69^b^
(+)-pinoresinol	1.56^a^	1.32^b,c^	1.12^c^	1.06^a^	0.90^a^	1.02^a^
Apigenin	3.28^a^	2.56^b^	2.23^b^	1.99^a^	1.89^a^	1.63^b^
Total	550.2^a^	523.0^b^	430.1^c^	520.3^a^	420.6^b^	350.3^c^

Values are the means of three analyses. Tukey´s test has been used to assess significance. Different letters in the same row indicate significant differences (p<0.05). Nd: values not detected; RI: ripening index

As far as concentrations of flavones and lignans are concerned, luteolin, apigenin and (+)-pinoresinol were more abundant in Arbequina and Hojiblanca varieties. There are studies on the phenolic content of olive fruits that reported an increase in luteolin concentrations and a decrease in apigenin concentrations with increasing ripeness, which is in accordance with the changes found in flavonoid concentrations in this study [42].

### Effect of olive oil phenolic compounds extract on MG-63 osteoblast cell proliferation

**Fig 1:**
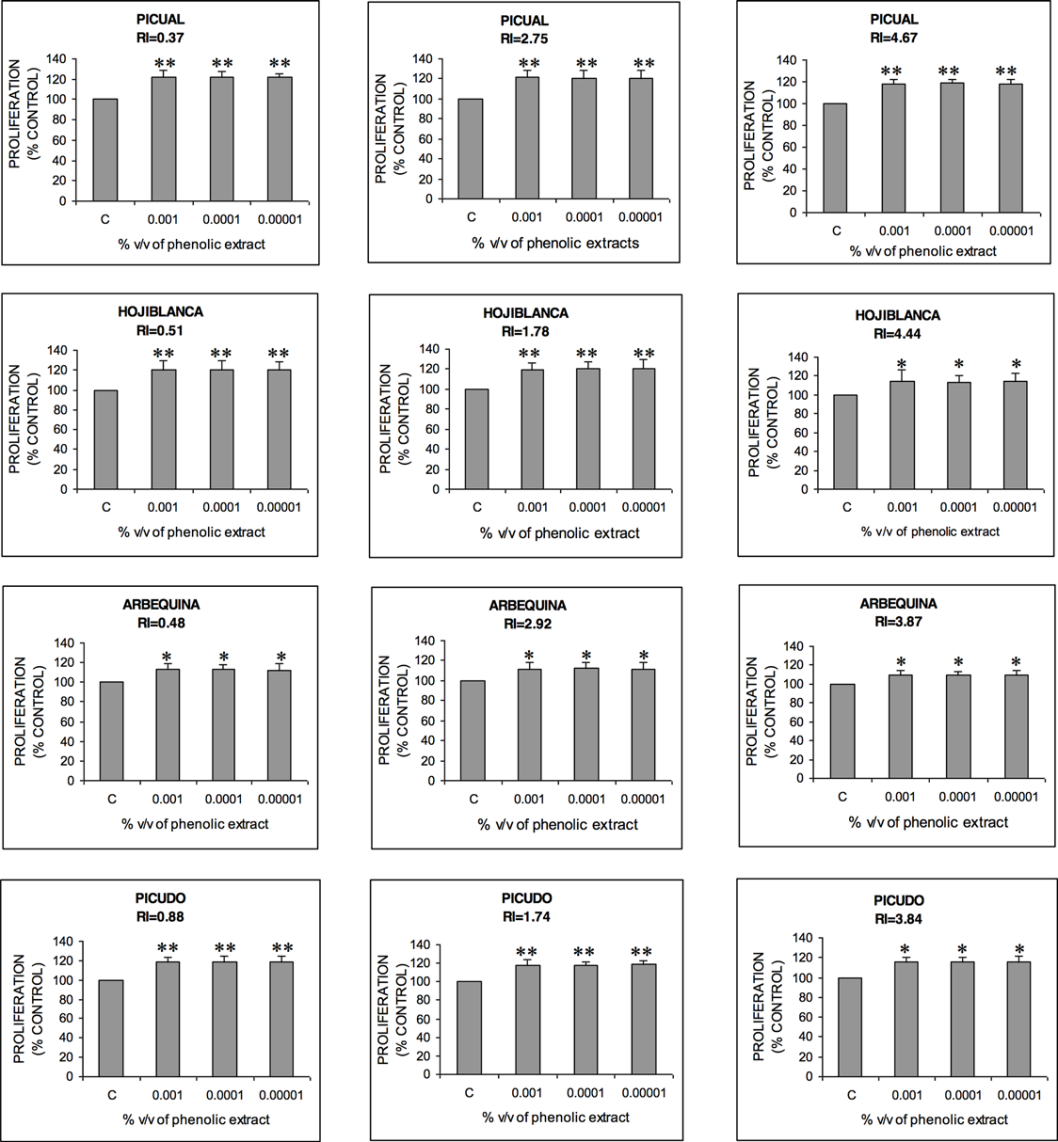
Effect of EVOO phenolic extracts on the growth of MG-63 cells as determined by MTT. Cells were treated with various concentrations of EVOO phenolic extract or vehicle alone (control) for 24 h. Data are means, with standard error of the mean shown by vertical bars of three separate experiments and at least every experiment was performed in triplicate. Mean values were significantly different as compared with control groups: *p<0.05, **p<0.01.

Recently, we have demonstrated the effects of VOO phenolic extracts obtained from Sicilian monovarietal olive oils on the proliferation of human osteosarcoma cells [9]. Treatment of osteoblast cells with Sicilian olive oil phenolic extracts increased the number of cells 13.77–30.98% compared with controls. In this work, the effect of EVOO phenolic extracts obtained from the most common four Spanish movarietal olive oils at three different ripening stages on the proliferation of human osteosarcoma cells was investigated. We hypothesized that the phenolic fractions of each EVOO variety may induce osteoblast proliferation differently. MG-63 cells were treated with various concentrations of extracts to determine the biological actions of complex multicomponent phenolic compounds. All extracts significantly stimulated MG-63 cell growth in a non-concentration dependent manner in this order of potencies: Picual> Hojiblanca> Picudo> Arbequina (Fig 1). The most effective EVOO phenolic extracts were those obtained from the Picual variety, as they significantly increased cell proliferation by 18–22%. Conversely, Arbequina phenolic extracts increased cell proliferation by 9–13%. It should be noted that Picual and Hojiblanca phenolic extracts–which have higher phenol concentrations–were more effective in inducing cell proliferation than the other varieties. Moreover, in all studied varieties there is a decrease in the ability to stimulate osteoblast proliferation in oils obtained from olive fruits collected at the end of the harvest, as total phenol content decreased in all four varieties.

The increase of cell proliferation induced by phenolic extracts was higher than that obtained by individual phenolic compounds. These results indicate that phenolic compounds in EVOO may synergize together to increase MG-63 cell proliferation to a greater level than a single treatment using individual chemicals. Although the observed effects suggest that there may be a synergism among phenolic compounds in EVOO extracts, other substances present in this extract can also cause MG-63 cell proliferation. However, there are not studies available that address the effect of other olive oil bioactive compounds on osteoblastic cell proliferation. Further studies are required to identify other bioactive substances and the mechanisms by which EVOO phenolic extracts induce MG-63 cell proliferation.

To date, the most consistently followed approach to examine the potential relation between EVOO phenolic compounds and bone health was based on particular phenols. The analysis considering the effect of a few isolated compounds on bone health misses information regarding complex or cumulative correlations and interactions between these compounds contained in olive oil. In this regard it should be considered that *in vitro* studies on individual phenolic compounds should always consider the effect of olive oil active fractions that could answer some questions and promote the development of appropriate recommendations for overall dietary habits.

Although these experimental studies support the hypothesis of EVOO phenolic compounds exert a beneficial effect on bone, further studies assessing the *in vivo* accessibility of EVOO phenolic compounds to osteoblast cells should be performed. Accordingly, and as EVOO phenolic compounds metabolism is still unknown, it is necessary to perform further *in vitro* studies where the intestinal absorption and biotransformation of biocompounds are considered. While other studies have revealed that olive oil intake results in increased urine and plasma concentrations of tyrosol and hydroxytyrosol [43] more research should be conducted to determine the bioavailability of other olive oil phenolic compounds.

### Statistical Analysis

**Fig 2:**
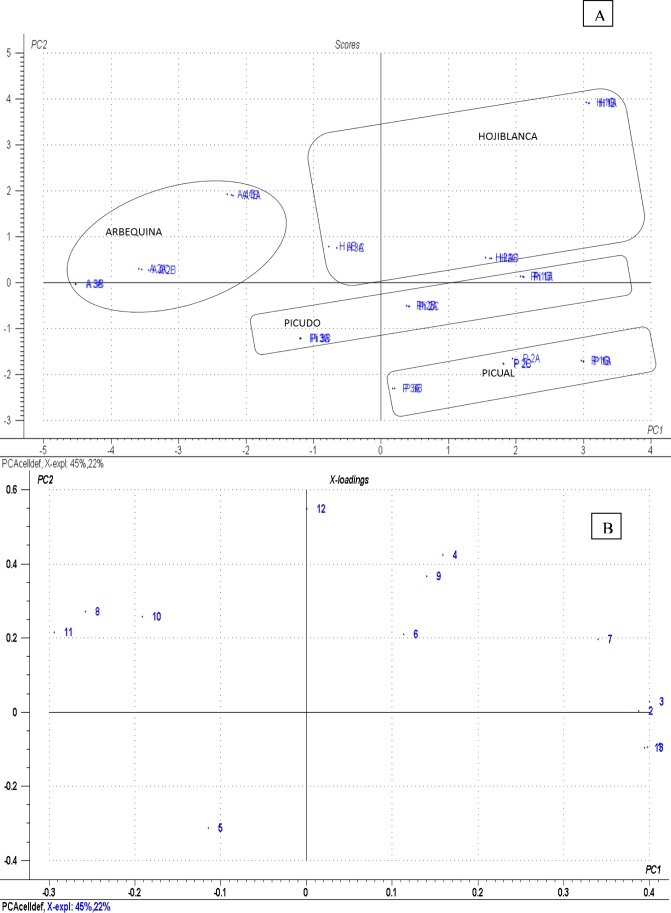
Bi-plot (A. Scores and B. Loadings) for the PCA model. Samples are plotted by selected letters and numbers: Picual RI 0.37 (P1); Picual RI 2.75 (P2); Picual RI 4.67 (P3); Hojiblanca RI 0.51 (H1); Hojiblanca RI 1.78 (H2); Hojiblanca RI 4.44; Arbequina RI 0.48 (A1); Arbequina RI 2.92 (A2); Arbequina RI 3.67 (A3); Picudo RI 0.88 (Pi1); Picudo RI 1.74 (Pi2); Picudo RI 3.84 (Pi3). The capital letters A, B and C correspond with 0.001, 0.0001 and 0.0001% extract concentration in osteoblast culture medium.

Principal component analysis was performed to explore data distribution patterns and visualize potential relationships among the study variables. Fig 2 shows the bi-plot graph for the Scores (A) and Loadings (B) obtained. The first-dimension PC-1 (which accounted for 45% of total variance) and the second dimension PC-2 (which accounted for 22% of variance) allowed us to distinguish the samples by olive variety and ripening stage. Table 4 shows the loading of the variables for the Principal Component 1 and 2. The values with the greatest factor loading for PC1 were MG-63 proliferation, total phenols, hydroxytirosol and tyrosol. Likewise, the variables showing a greater factor loading of the PC2 component were caffeic acid and apigenin. Comparative analysis of the two PCA plots, scores, and loading plot indicated that MG-63 proliferation and total phenols were the main factors for identifying Picual oils obtained from early-harvested fruits, while vanillic acid is related to oils obtained from Picudo variety. Conversely, the variables caffeic acid, vanillin and oleuropein differentiate Hojiblanca oils. In addition, the phenolic compounds tyrosol, luteolin, pinoresinol and ferulic acid were mainly responsible for the grouping of EVOO obtained from the Arbequina variety.

**Table 4:** X loadings for the variables with respect to the Principal Component 1 and 2.

	Variable	PC1	PC2
1	Proliferation	0.40	-0.09
2	Hydroxitirosol	0.39	0.00
3	Tyrosol	0.40	0.03
4	Caffeic acid	0.16	0.42
5	Vanillic acid	-0.11	-0.31
6	Vanillin	0.11	0.21
7	P-coumaric acid	0.34	0.20
8	Ferulic acid	-0.26	0.27
9	Oleuropein	0.14	0.37
10	Luteolin	-0.19	0.26
11	Pinoresinol	-0.29	0.22
12	Apigenin	0.0005	0.55
13	Total phenols	0.40	-0.10

## Conclusion

This study concludes that phenolic compounds and extracts from different extra virgin olive oil varieties stimulate osteoblast cells (MG-63) proliferation. Further research on the signaling pathways of olive oil phenolic compounds involved in the processes and their metabolism should be carried out to develop new interventions and adjuvant therapies using EVOO for bone health (i.e. osteoporosis) in adulthood and the elderly.
